# Integrating longitudinal mental health data into a staging database: harnessing DDI-lifecycle and OMOP vocabularies within the INSPIRE Network Datahub

**DOI:** 10.3389/fdata.2024.1435510

**Published:** 2024-10-11

**Authors:** Bylhah Mugotitsa, Tathagata Bhattacharjee, Michael Ochola, Dorothy Mailosi, David Amadi, Pauline Andeso, Joseph Kuria, Reinpeter Momanyi, Evans Omondi, Dan Kajungu, Jim Todd, Agnes Kiragga, Jay Greenfield

**Affiliations:** ^1^African Population and Health Research Center (APHRC), Nairobi, Kenya; ^2^Strathmore University Business School, Strathmore University, Nairobi, Kenya; ^3^Department of Population Health, London School of Hygiene and Tropical Medicine, London, United Kingdom; ^4^Artificial Intelligence and Machine Learning (AI and ML), CODATA-Committee on Data of the International Science Council, Paris, France; ^5^Institute of Mathematical Sciences, Strathmore University, Nairobi, Kenya; ^6^Iganga Mayuge Health and Demographic Surveillance Site (IMHDSS), Makerere University Centre for Health and Population Research (MUCHAP), Kampala, Uganda; ^7^Infectious Diseases Institute, College of Health Sciences, Makerere University, Kampala, Uganda

**Keywords:** longitudinal mental health, OMOP Common Data Model, DDI-lifecycle, staging database, extract, transform and load

## Abstract

**Background:**

Longitudinal studies are essential for understanding the progression of mental health disorders over time, but combining data collected through different methods to assess conditions like depression, anxiety, and psychosis presents significant challenges. This study presents a mapping technique allowing for the conversion of diverse longitudinal data into a standardized staging database, leveraging the Data Documentation Initiative (DDI) Lifecycle and the Observational Medical Outcomes Partnership (OMOP) Common Data Model (CDM) standards to ensure consistency and compatibility across datasets.

**Methods:**

The “INSPIRE” project integrates longitudinal data from African studies into a staging database using metadata documentation standards structured with a snowflake schema. This facilitates the development of Extraction, Transformation, and Loading (ETL) scripts for integrating data into OMOP CDM. The staging database schema is designed to capture the dynamic nature of longitudinal studies, including changes in research protocols and the use of different instruments across data collection waves.

**Results:**

Utilizing this mapping method, we streamlined the data migration process to the staging database, enabling subsequent integration into the OMOP CDM. Adherence to metadata standards ensures data quality, promotes interoperability, and expands opportunities for data sharing in mental health research.

**Conclusion:**

The staging database serves as an innovative tool in managing longitudinal mental health data, going beyond simple data hosting to act as a comprehensive study descriptor. It provides detailed insights into each study stage and establishes a data science foundation for standardizing and integrating the data into OMOP CDM.

## 1 Introduction

In the contemporary landscape of global mental health research, the need for precise measurement and effective management of mental health disorders has never been greater, particularly within the dynamically diverse settings of Africa. Mental health challenges have been intensified by socioeconomic disparities, stigma, and sub-optimal healthcare infrastructures, expanding the imperative for extensive and incisive research (Kiwuwa-Muyingo et al., 2023). Historically, the focus of healthcare policy in Africa has been predominantly on infectious diseases; however, the growing impact of mental health conditions calls for an evolution in data collection and analytical methods. The scarcity of standardized mental health data within the continent notably impedes the progress of developing refined interventions and policies.

The increasing impact of mental health conditions in Africa necessitates a shift toward more precise measurement and effective management. This includes an emphasis on the evolution of data collection and analytical methods to address the continent's scarcity of standardized mental health data, which hinders the development of refined interventions and policies (OHDSI, a; Bhattacharjee et al., 2024).

The Observational Medical Outcomes Partnership (OMOP) Common Data Model (CDM) provides a framework for organizing healthcare data from diverse sources into a standardized format, enabling robust analyses across different datasets and countries (Levene and Loizou, 2003). At the core of this initiative is establishing a staging database, following international metadata standards, to transform data into the OMOP CDM. The staging database is structured as a snowflake schema to capture the complexities and nuances of a population undergoing constant change in a longitudinal study (Planche et al., 2022; Nicholas et al., 2022). It also serves as a gateway for integrating place-based exposures like Sustainable Development Goal (SDG) implementation indicators, social determinants, and environmental exposures for further analysis, offering a holistic view of the factors influencing mental health outcomes (Deguen et al., 2022). All questions, answers, variables, and values in the staging database are semantically annotated using standard international vocabularies or continent-specific concepts, providing comprehensive details about the study population and its impact on mental health.

The INSPIRE MH Project aims to evaluate the mental illness burden and map available community resources for alleviating this burden by harnessing primary and secondary data from ongoing and prior longitudinal studies. This comprehensive model serves as a dynamic framework to capture the interplay between the prevalence of mental health issues and the efficacy of community support structures (Moeti, 2022; Duda et al., 2020). The utilization of the staging database in this study is paramount, emphasizing its role in transforming both primary and secondary collected data into a harmonized, standardized format for subsequent analysis (Essock et al., 2015). Integrating socio-behavioral and environmental data provides a comprehensive framework for analyzing the multifaceted dimensions of health outcomes.

From the utilization of the staging database, this paper offers significant contributions to mental health research, particularly in Africa. Firstly, it establishes a robust staging database that facilitates harmonizing, transforming, and analyzing longitudinal mental health datasets. This database ensures high data quality and consistency, serving as a central repository for diverse data sources. Secondly, the project develops and implements a comprehensive mapping strategy that aligns these datasets with the OMOP CDM requirements. This approach standardizes data formats and terminologies, enabling seamless integration and analysis across different datasets and institutions. Furthermore, the project incorporates local concepts to build relevant vocabularies for use in OMOP and other data analysis workbenches. This effort enhances the contextual relevance and applicability of the data for regional analyses, making the findings more meaningful and actionable. Finally, the project evaluates the effectiveness of the staging database in mapping longitudinal mental health datasets, demonstrating its potential for broader adoption across various health conditions. By enhancing data harmonization, standardization, and usability, the paper provides a scalable and generalizable model that can be adopted by other researchers and institutions, ultimately informing better mental health interventions and policies.

This paper is organized based on an analysis of the existing methods applied to standardize longitudinal mental health data. It is structured as follows: Section 2 covers related works, Section 3 provides the background of the study, Section 4 is the proposed methodology for the current research, Section 5 covers experiment and results, Section 6 covers the discussion, Section 7 covers conclusions, and Section 8 depicts future work.

## 2 Related works

Several notable efforts have been made to address the challenges of data collection, anonymization, standardization, and analysis using data science techniques in mental health research, particularly within the African context. This section reviews related works that have contributed to advancing methodologies and frameworks for mental health data management and their application in improving mental health outcomes.

### 2.1 Longitudinal studies on mental health disorders

Longitudinal studies have been pivotal in understanding the progression and impact of mental health conditions over time. Research (Kessler et al., 2005) utilized longitudinal data to investigate the lifetime prevalence of DSM-IV disorders using data from the National Comorbidity Survey. This study underscores the importance of capturing data over extended periods to identify trends and inform interventions. However, the challenge of integrating and analyzing longitudinal data remains, particularly in resource-limited settings like many African countries.

### 2.2 Standardized data collection and integration

One significant effort in standardizing health data collection is the development of the Observational Medical Outcomes Partnership (OMOP) Common Data Model (CDM). The OMOP CDM provides a standardized framework for organizing healthcare data from diverse sources, enabling robust and comparative analyses across different datasets and countries (Vardigan et al., 2008). This model has been widely adopted in various healthcare research initiatives, demonstrating its utility in harmonizing complex datasets and facilitating large-scale epidemiological studies.

### 2.3 Metadata standards and semantic annotation for big data management

International metadata standards, such as the Data Documentation Initiative (DDI) Lifecycle specifications, have facilitated the systematic documentation and management of research data. Studies by Vardigan et al. (2008) and Greenfield and Carpenter (2013) discuss the benefits of employing these standards in enhancing data quality, interoperability, and reusability. Semantic annotation of data, using standardized vocabularies, further enriches the data, making it more accessible and interpretable for various research purposes.

### 2.4 Data harmonization and interoperability

Efforts to harmonize longitudinal health data across different regions and systems have been critical in advancing global health research. The 20-year study on analyzing the global burden of disease (Murray, 2022) is an example of a large-scale project that integrates data from multiple sources to comprehensively assess health outcomes worldwide. This approach demonstrates the feasibility and benefits of data harmonization in producing reliable and comparable health metrics.

### 2.5 Data integration frameworks

The star schema, introduced by Kimball (1996), has been a foundational model for organizing and querying data in various domains. Building upon this, the Informatics for Integrating Biology and the Bedside (i2b2) data mart adopted Kimball's star schema to represent, host, query, and analyze patient biomedical data collected in hospital settings i2b2 (Healthcare, 2017).

**Figure 1:**
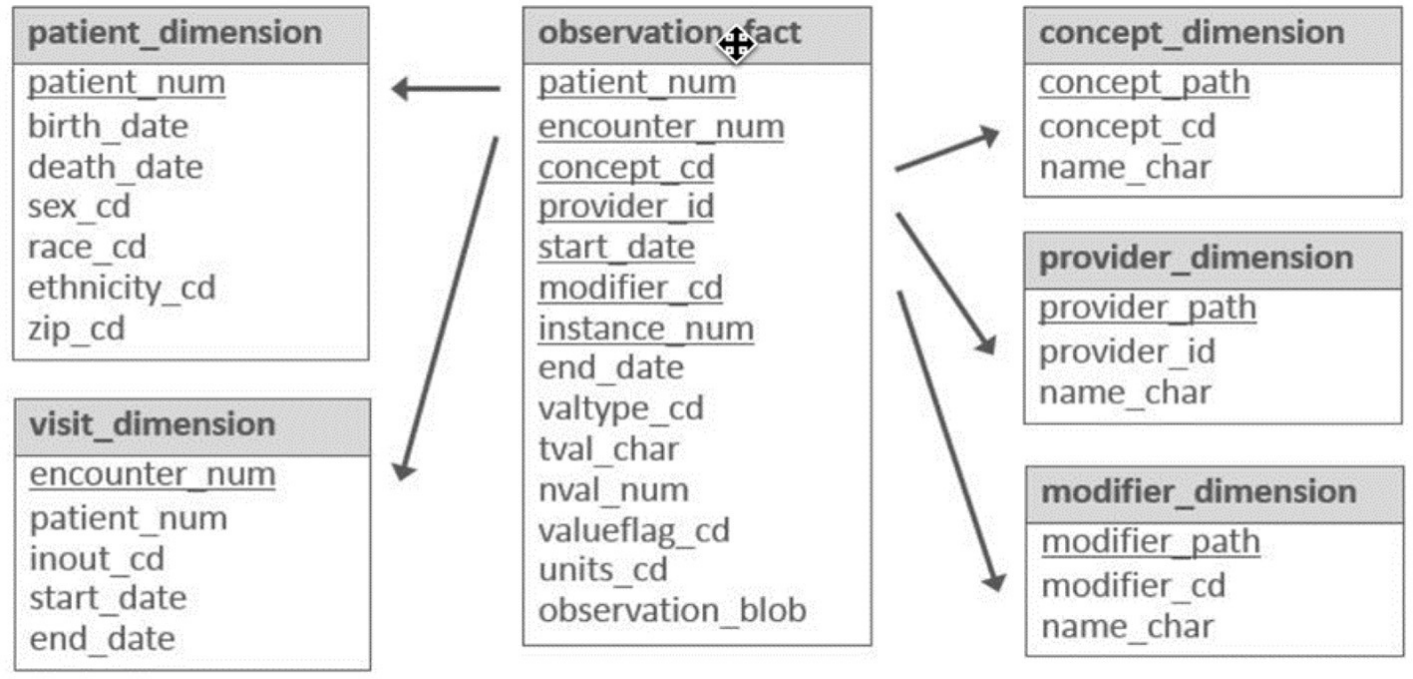
Main tables and fields in the i2b2 star schema.

Figure 1 illustrates the main tables and fields in the i2b2 star schema. DDI Data Documentation 3.3 is a standard for describing data collected in longitudinal studies, although there isn't a comparable star schema for hosting population health longitudinal studies. In DDI 3.3, encounters occur in questionnaire-specific interviews and tests repeated over time, with only interviewers and no providers. A Variable in DDI 3.3 corresponds to an observation fact in i2b2 and records an observation or a measurement along with its context.

One concern is the potential loss of information when translating data from the staging database to common data models that capture clinical care. This highlights the need for a common data model capable of managing mental health data from community and clinical care settings. Initiatives like the Bridge2AI movement, which aims to construct new flagship biomedical and behavioral datasets that are ethically sourced, trustworthy, well-defined, and accessible, could facilitate this need.

By integrating these comprehensive data models and standards, the staging database developed in this work aims to bridge the gap between community-based mental health data and clinical care data, enabling robust and holistic analyses that inform better mental health interventions and policies (NIH).

## 3 Background of study

Historically, public health policymakers in Africa have focused on communicable diseases, such as malaria, tuberculosis, and HIV/AIDS. However, non-communicable diseases, such as cancer, heart disease, diabetes, and mental health conditions are increasingly becoming the main cause of mortality in Africa. According to the World Health Organization (WHO), across the region, an estimated 116 million people were living with mental health disorders pre-pandemic. Coronavirus disease-2019 (COVID-19) has exacerbated mental health conditions due to unexpected deaths, lack of social interactions, school shutdowns, and economic decline, which have led to an estimated 25% global increase in the prevalence of depression and anxiety (Aghababaie-Babaki et al., 2023).

There is a continuous need for harmonized data on mental health in African settings to assist in improving the appropriateness and accuracy of mental health screenings and to develop a more nuanced and culturally appropriate mental health database. Data on the causes, consequences, and impacts of mental health conditions in the African population, therefore, needs to be collected and harmonized. These data needs to capture and account for physical exposures, socio-economic forces, health opportunities, and lifestyles, often referred to as the external exposome (Safarlou et al., 2023), which impact mental health. This necessitates data from longitudinal research, which could also show the effectiveness of interventions to improve mental health in the region.

Research on mental health in has primarily centered on identifying the causes of conditions that are specific to African contexts, developing low-cost interventions to help those affected, and monitoring the impact of mental health services on those receiving them. Harmonizing longitudinal mental health data using multiple sources will facilitate evidence-based decisions about mental health conditions and allow comparisons that enhance or impede effective treatment. These factors collectively affect the ability to meet the increasing demand for accurate, accessible, up-to-date mental health data, which in turn negatively affects evidence-based decision-making, policy formulation, and service delivery in the mental health sector. This research will address these critical gaps, thereby enhancing the understanding and management of mental health in Africa (Safarlou et al., 2023).

## 4 Methods

**Figure 2:**
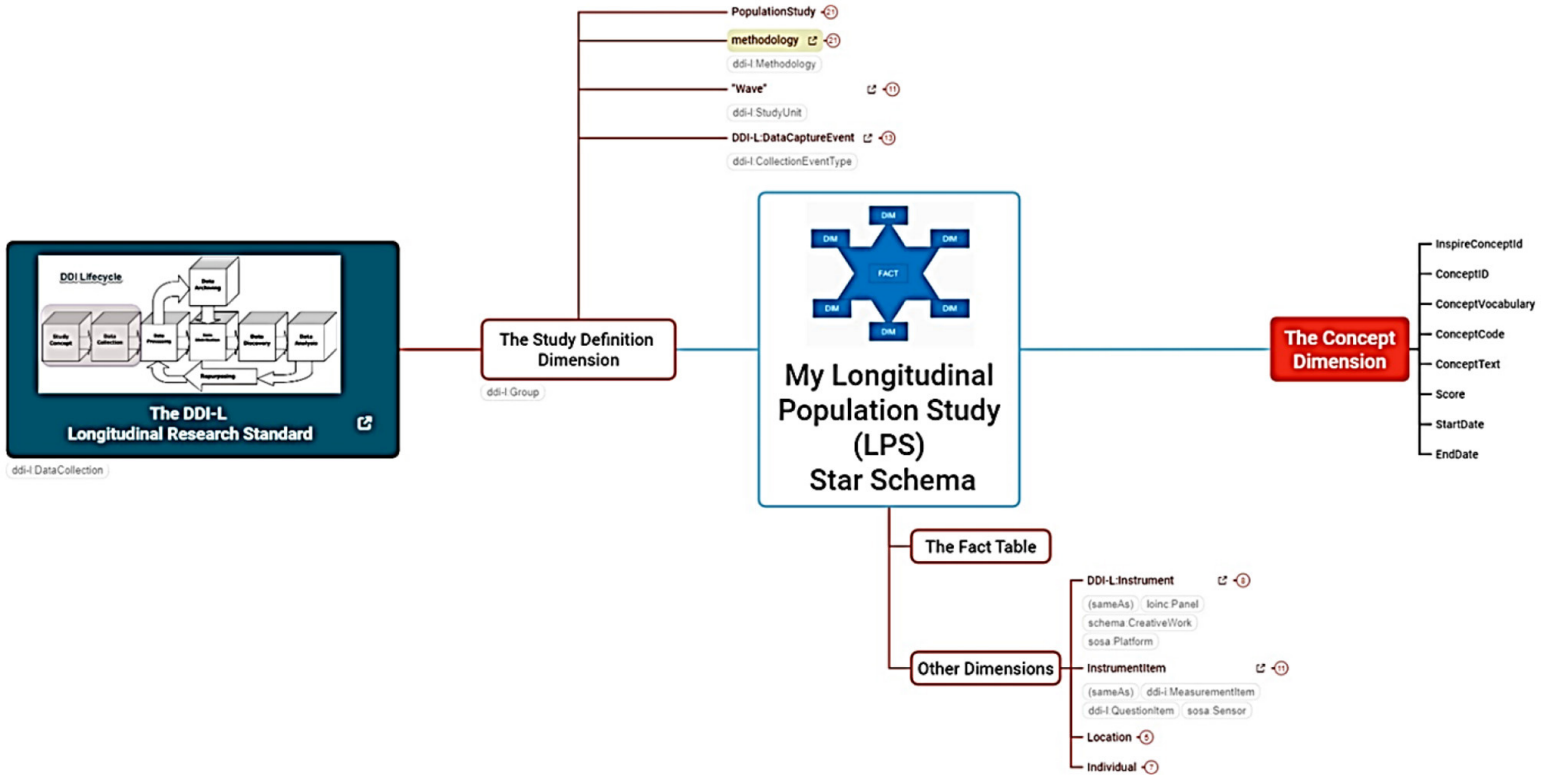
The INSPIRE project mind map. The detailed mind map link can be accessed here.

The staging database is designed using the DDI Lifecycle to manage data across the entire lifecycle from conceptualization to publication and beyond. It improves the ability to capture metadata at its point of origin and then track its use and change over time, as elaborated in Figure 2.

### 4.1 Overview of project mind map

The database follows a snowflake schema data warehouse model, with a centralized FACT table (LPS Fact) connected to multiple dimensions in normalized form across related tables.

#### 4.1.1 Fact

A central table that stores actual data in codes derived from the concept table, including relationships to individuals, instruments, locations, and household data. Data is mapped using the source study's OMOP concept codes and local concepts.

#### 4.1.2 Metadata (study definition dimension)

This dimension stores details such as data source, data type, date of acquisition, and transformations, aligning with the DDI Lifecycle. One key feature is the reuse of metadata throughout the data life cycle to avoid duplication of efforts and ensure findability.

#### 4.1.3 Concepts dimension

Stores concepts equivalent to the OMOP model, including mental health concepts mapped as INSPIRE LOCAL CONCEPTS. Figure 2 depicts INSPIRE's mental health project using a mind map.

The description of the mind map illustrated in Figure 2 is provided in Table 1.

**Table 1:** The descriptions of the components used in the mind map.

**No**	**Table**	**Description**
1	The DDI-L longitudinal research standard (bottom left)	This box represents the DDI-L standards, which serve as a format for documenting and managing data across its lifecycle and organizing study data
2	The study definition dimension (in red, connected to the DDI-L)	Defines the study methodology, data collection waves, and study groups in a structured manner
3	My longitudinal population study (LPS) snowflake schema (center)	The schema is optimized for data warehousing and reporting. A FACT table stores quantitative data from mental health datasets, and other dimensions represent qualitative and descriptive attributes.
4	The FACT table (in red, connected to the snowflake schema)	The FACT table serves as the core of the snowflake schema, connecting to various other dimensions.
5	Other dimensions (in red, connected to the FACT table)	These include instruments, measurement items, locations, and individuals, supporting multi-dimensional analysis capabilities.
6	The concept dimension (to the right)	This dimension handles the metadata related to concepts, including concept IDs, vocabularies, codes, texts, and temporal aspects. It is essential for harmonizing and transforming data into analysis workbenches.

### 4.2 Schema design

The schema design includes standardized staging vocabulary, metadata, individual, and FACT tables, each serving specific data organization and management roles. The design aims to streamline data organization, enhance data quality, and support rigorous analysis in longitudinal population studies. The standardized staging FACT table and the LONGITUDINAL_POPULATION_STUDY_FACT table act as the central repository for longitudinal study data, linking individual-level data to specific interviews, resident episodes, population studies, instrument items, concepts, and value types, enabling efficient data management, analysis, and interpretation. This comprehensive schema design aims to streamline data organization, enhance data quality, and support rigorous analysis in longitudinal population studies (Voss et al., 2015).

### 4.3 Developing the schema and staging database

In developing the staging database, the logical snowflake schema was translated into a functional database, involving the definition of the storage schema, table structure, and implementation of security measures. These measures are essential for ensuring data integrity and security. The initial logical schema for longitudinal mental health data and metadata was turned into a script using SQL commands, exported, and then executed in the DBMS to create tables and enforce constraints for data integrity (Kajungu et al., 2020).

The staging database plays two key roles in the research framework. It is a transparent portal for ongoing study execution and forms the foundation for all reporting activities. Additionally, it is designed for adaptability, enabling the migration of studies into the OMOP CDM through an ETL process. This capability facilitates various descriptive, predictive, and causal analyses. Notably, within the ATLAS platform, the database integrates with advanced analytical methodologies to enhance the study of treatment outcomes and impacts within healthcare research.

The staging database contains multiple tables alongside the central FACT table, each with essential metadata that enhances data interpretability. For example, the “wave” table uses the “instrument_model_type” attribute to ensure consistency through a controlled vocabulary. The database accommodates different gender concepts and captures data nature through attributes like “gender” and “instrument item type.” The term “facts” refers to empirical data from instrument items in longitudinal population studies. The text explores optimizing metadata handling in “dimension” tables for improved data analysis in these studies. The ensuing sections delve into this aspect, highlighting the approaches used and the resulting implications for data analysis in longitudinal population studies.

#### 4.3.1 Description of the dual-endpoint model

Using a dual-endpoint model, incorporating both a staging database and OMOP CDM, played a crucial role in the successful longitudinal harmonization of mental health data. The staging database was instrumental in capturing the entire study's execution, ensuring data mapping and interpretation consistency across different phases of the harmonization process (Holistics).

**Figure 3:**
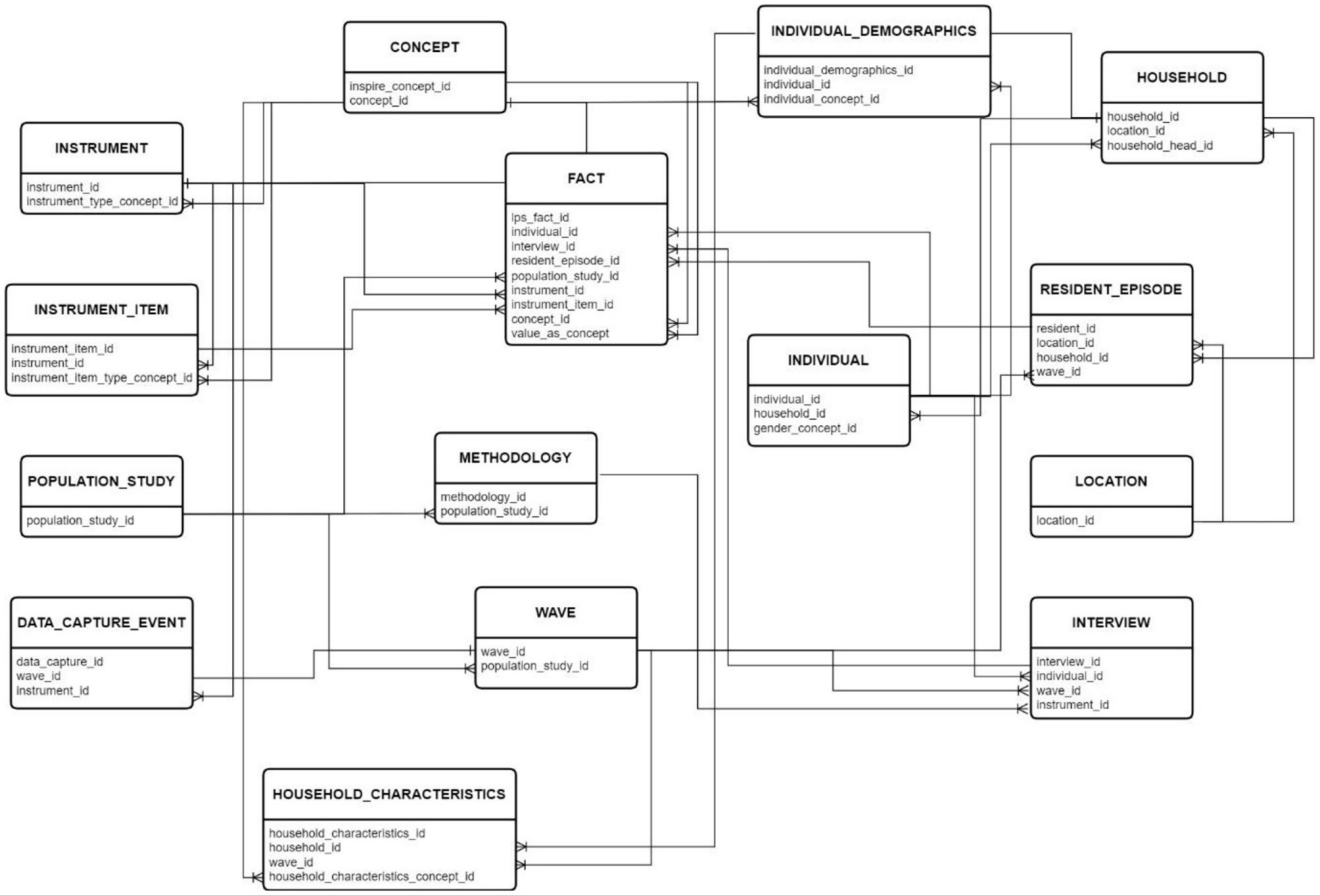
The detailed schema can be accessed here.

Figure 3 presents the Entity-Relationship Diagram (ERD) depicting the database's relational design, laying the groundwork for implementing a snowflake schema within the staging database. The ERD contains only the key fields to show the table's relationship. The sections below illustrate a breakdown of each section of the tables.

**Figure 4:**
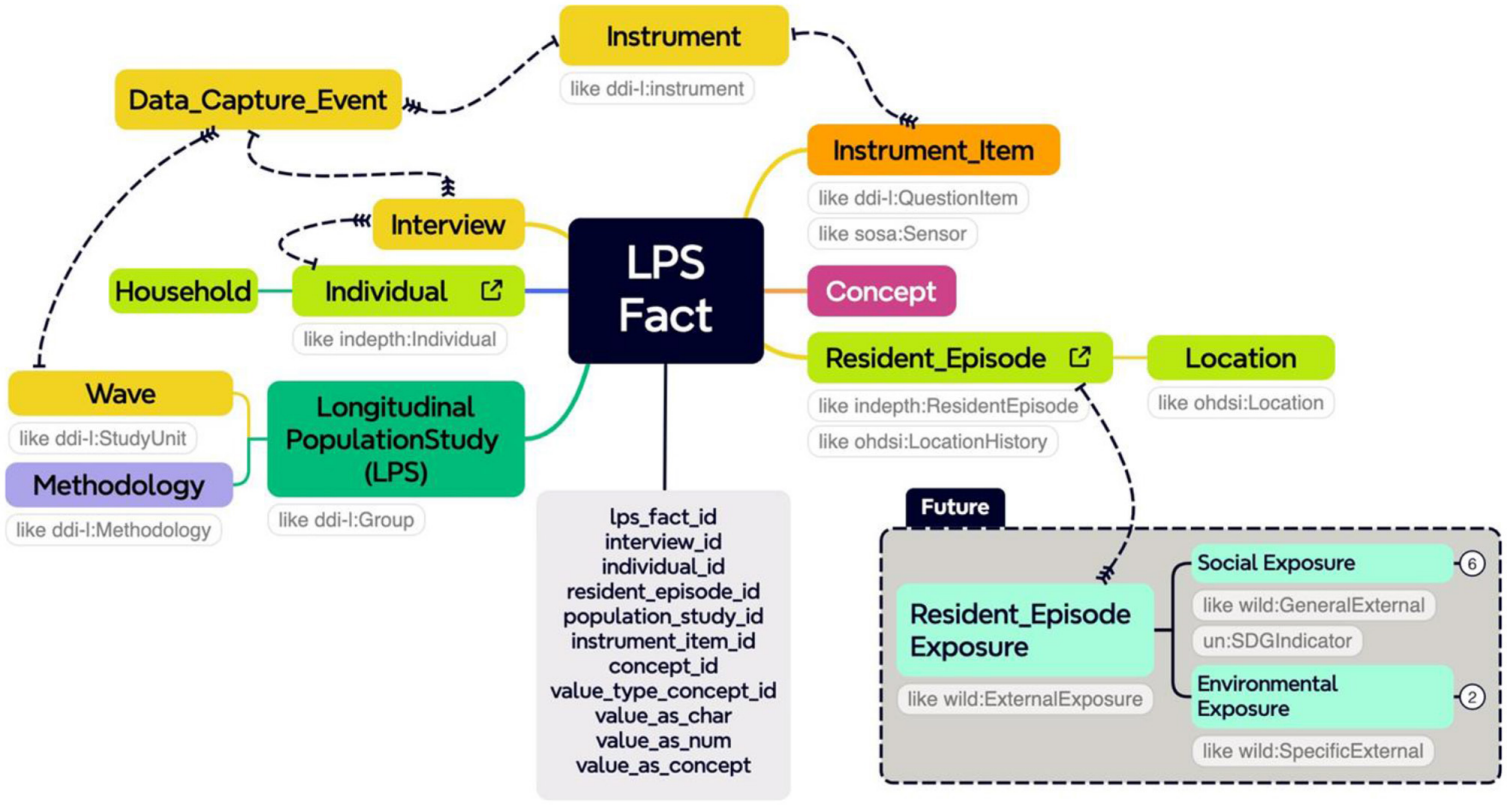
The model link can be accessed here.

Figure 4 is the dual end-point model. It provides a strategic framework that streamlines both upstream and downstream processes essential for the development and population of a staging database.

## 5 Experiments and results

The workflow outlines a structured sequence of transformations essential for migrating longitudinal mental health data into the INSPIRE staging database using Pentaho Data Integration software. It involves loading the initial dataset into a PostgreSQL database, mapping metadata, and integrating OMOP CDM vocabularies. Unique local Concept IDs are assigned within the staging database for data concepts lacking equivalent terms in the OMOP framework (Community.i2b2).

### 5.1 Mapping metadata into the staging database

Gathering essential information from longitudinal studies, documenting each data collection wave, and integrating metadata into the PostgreSQL database streamlined data operations and laid the groundwork for comprehensive analysis and reporting.

The metadata tables contain essential information about study designs, methodologies, data collection events, and other relevant details. The POPULATION_STUDY table stores information about mental health status investigations, the METHODOLOGY table outlines data collection approaches, the WAVE table provides an overview of data collection phases, and the DATA CAPTURE EVENT table summarizes data capture occasions.

**Figure 5:**
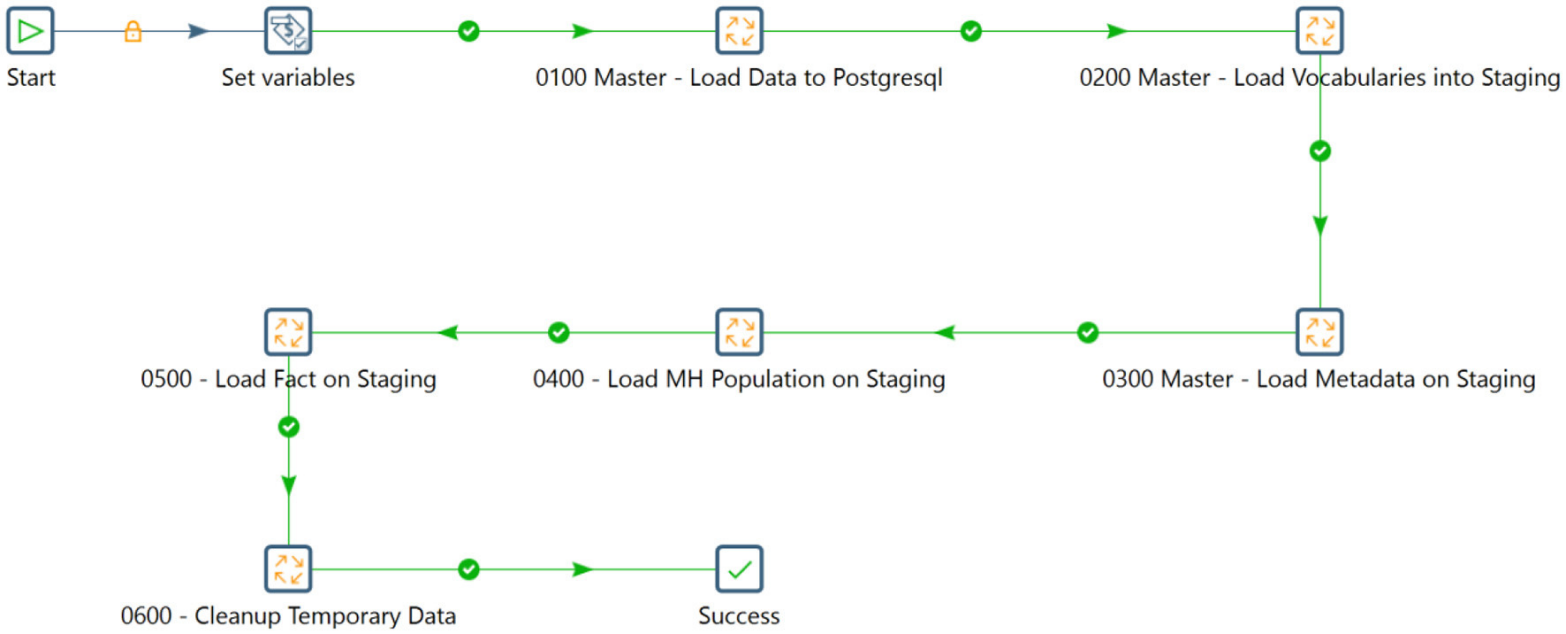
The staging workflow.

Figure 5 illustrates the workflow for transferring metadata from the source mental health tools to the INSPIRE staging database.

### 5.2 Mapping the data into the staging database

Mapping datasets into a staging database is an essential step in the data integration. It facilitates the transition of raw data or source data into a structured environment for further processing and analysis. Utilizing a practical use case to illustrate this process provides valuable insights into its significance and implementation.

#### 5.2.1 Use case: longitudinal, population-based data

A mental health survey conducted in Iganga-Mayuge in 2023 was chosen as a use case to demonstrate the implementation of this strategy. The survey collected data using the Patient Health Questionnaire (PHQ-9), which is used to screen for depression using nine questions related to symptoms of depression. General Anxiety Disorder (GAD-7), which is a screening tool for anxiety and contains seven questions, and the 24-item Behavior and Symptom Identification Scale (BASIS-24), which includes 24 questions aiming to get the severity of psychosis symptoms in the target cohort.

#### 5.2.2 The database designing tool

A data modeling tool is essential for creating and managing data models and representing data structures visually. We used dbdiagram.io, an online collaborative tool with a user-friendly interface, to design and visualize database schemas.

#### 5.2.3 The data integration tool

Pentaho Data Integration (Hitachi Vantara), or Kettle, is a versatile tool for data transformation and organization. Owned by Hitachi Vantara, it offers a free community edition that is accessible to all users. Its intuitive graphical interface enabled us to design complex data workflows effortlessly through drag-and-drop components, expediting development and deployment.

#### 5.2.4 The mapping process

Mapping is the systematic alignment of data elements from various sources into a standardized format, the goal of which is to establish correlations, define relationships, and ensure coherence in structure and content. The mapping process systematically aligns data elements from different sources into a standardized format. The goal is to establish correlations, define relationships, and ensure consistency in both structure and content. Mapping is essential for efficient data governance, discovery, and utilization. In this use case, the workflow is carefully designed to optimize data progression and ensure smooth transformation into the desired structure within the staging database. This meticulous design facilitates seamless data flow and efficient navigation through all necessary stages to achieve alignment with the target data structure.

#### 5.2.5 The vocabulary tables

The vocabulary was loaded onto the staging database to standardize data and ensure consistency during integration. This allowed data analysts to align sources using a common framework, enabling accurate analysis. Key vocabulary tables, like CONCEPTS, INSTRUMENT, and INSTRUMENT_ITEM, were crucial within the staging dataset, providing a comprehensive repository of concepts and assessment tools. The structured approach enabled easy identification of assessment tools used across studies and provided insights into their usage. Additionally, user-defined concepts were incorporated into the staging database to facilitate seamless mapping and potential integration of missing concepts into the standard vocabulary in the future (Hitachi Vantara).

This proactive approach ensured comprehensive data coverage and paved the way for ongoing enhancement and refinement of the data mapping process within the INSPIRE mental health data ecosystem.

#### 5.2.6 The population and survey data

Loading population and survey data is pivotal in integrating data within the INSPIRE mental health data ecosystem. This involves transferring demographic information and survey responses from various sources into the staging database. Key tables within the staging database include INDIVIDUAL, INDIVIDUAL_DEMOGRAPHICS, HOUSEHOLD, HOUSEHOLD_CHARACTERISTICS, RESIDENT_EPISODES, and INTERVIEW. The INDIVIDUAL table serves as a repository for storing unique identifiers associated with individuals, maintaining constant values throughout an individual's lifespan. Conversely, the INDIVIDUAL_DEMOGRAPHICS table is designed to accommodate various parameters or values related to individuals in a relational format, utilizing concepts from the Vocabulary section. The connection between these two tables allows for dynamic allocation of demographic features to individuals, ensuring flexibility and efficiency in managing individual-level data while maintaining relational integrity.

The HOUSEHOLD table is a unique identifier for each surveyed household, capturing location details through GPS coordinates for precise tracking within the survey dataset. The HOUSEHOLD_CHARACTERISTICS table stores diverse parameters and values related to households in a relational format, drawing upon concepts from the Vocabulary section. This setup enables the dynamic connection of households with various parameters, such as income details, and streamlines data management.

The RESIDENT_EPISODE table records the residential episodes of individuals within the surveyed population across different waves, providing crucial details about location, household, and wave. This offers insights into their living situations over time.

Finally, the INTERVIEW table stores details of interviews conducted during the survey, including dates, waves, and instruments used. This information provides context for the survey administration process and aids in understanding circumstantial events surrounding interviews. Including interview dates is essential for future migration to OMOP CDM (Data Documentation Initiative-CDI).

#### 5.2.7 The FACT table

Implementing a FACT table is crucial for constructing a mental health data warehouse. The FACT table stores quantitative data and key performance indicators related to mental health assessments, interventions, and outcomes. It allows for efficient and accurate analysis and facilitates querying, reporting, and deriving insights. Additionally, it serves as a stage for migrating data into the OMOP CDM. The LONGITUDINAL_POPULATION_STUDY_FACT table in the staging database is a central table, linking to various dimensional tables in a snowflake schema. It consolidates key metrics and observations from longitudinal population studies, providing a comprehensive view of trends, patterns, and outcomes over time.

#### 5.2.8 The extract transform and load process

**Figure 6:**
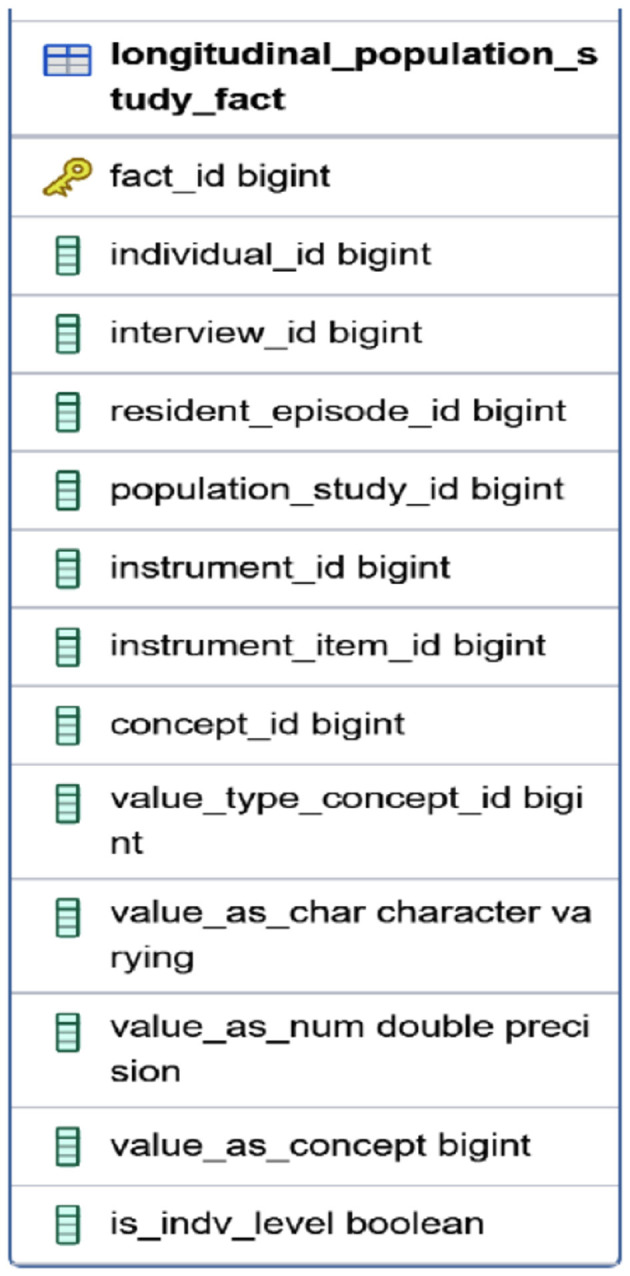
The FACT table.

**Figure 7:**
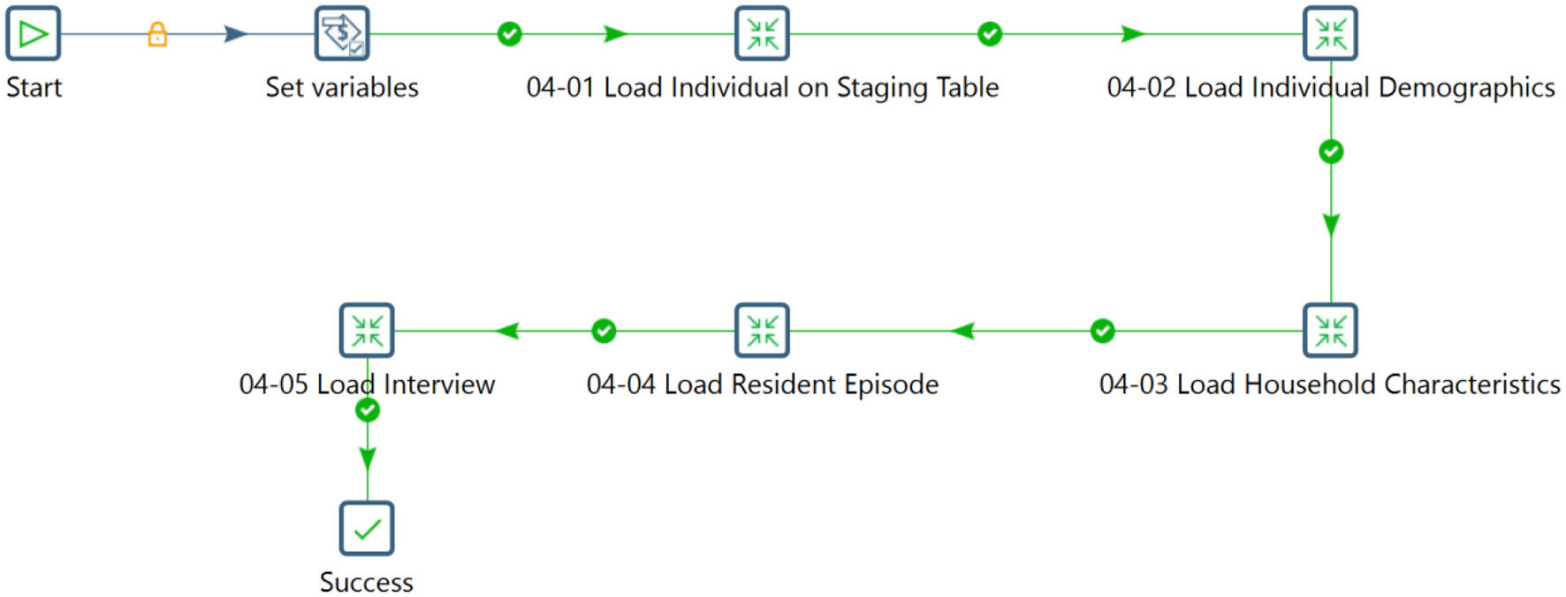
Flow for loading data into the population related tables.

**Figure 8:**
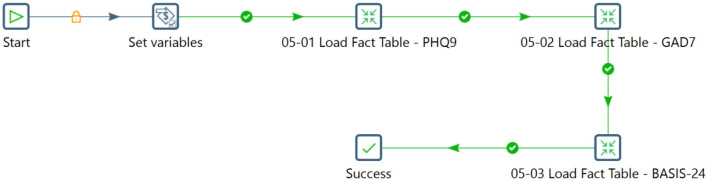
Sequencing the transformation within a job to load the fact table.

The ETL process integrates data from various sources, ensuring consistency and quality. It includes extracting data, transforming it to a consistent format, and loading it into a target destination. The Pentaho Data Integration tool was used in this study to streamline the data flow and handle complex operations effectively. Initially, components were linked to convert data to PostgreSQL format, ensuring data integrity and security. Individual and household data covering demographics, characteristics, episodes, and interviews were then integrated into the staging database. Figures 6–8 illustrates the flow of transformations loading population-related tables in the staging database (OHDSI, b).

The process involved transforming input data into a relational structure within the INDIVIDUAL table and systematically integrating the LOCATION and HOUSEHOLD tables. Complex steps populated data into the INDIVIDUAL DEMOGRAPHICS table and loaded data into the HOUSEHOLD_CHARACTERISTICS, RESIDENT_EPISODE, INTERVIEW, and FACT tables. The systematic arrangement categorized the data for subsequent analysis and interpretation.

To compile questions and responses from each participant for the PHQ-9, GAD-7, and BASIS-24 questionnaires, the transformation process extracted data from the newly populated INDIVIDUAL, INTERVIEW, RESIDENT_EPISODE, and INSTRUMENT_ITEM tables. This information was subsequently merged with the wide-format mental health survey data to produce comprehensive insights into the questionnaire's inquiries and participant responses. The resulting dataset was formatted longitudinally within the relational table dedicated to the questionnaires. The ETL process consolidated, transformed, and organized mental health survey data from the Iganga site into a structured format. Commencing with data extraction from spreadsheets and PostgreSQL databases, the process proceeded through stages of restructuring and mapping. Complex transformations were implemented to ensure compatibility with the staging database schema, preserving data integrity and quality. The staging database stands prepared to facilitate further processing, migration into OMOP CDM, comprehensive analysis, and reporting through the seamless integration of concepts, metadata, and individual, household, and survey data.

### 5.3 Results

A standardized representation of mental health data was achieved through comprehensive mapping and harmonization efforts. This standardization lays the foundation for analysis and future interoperability within the INSPIRE Network Datahub ecosystem. The Vocabulary set of tables integrated details of mental health assessment tools, utilizing OMOP vocabularies where available and introducing the INSPIRE vocabulary to accommodate values not covered by OMOP. Seven tools were recorded in the INSTRUMENT table, with specific details of each tool captured in the INSTRUMENT_ITEM table. Table 2 lists the mental health assessment tools incorporated into the repository (data warehouse dimension tables) for use across various population studies.

**Table 2:** The list of mental health assessment tools in the staging database.

**Instrument_id**	**Name**	**Description**
1	Patient Health Questionnaire (PHQ-9)	Screening tool for depression
2	Generalized Anxiety Disorder (GAD-7)	Screening tool for anxiety
3	Depression, Anxiety and Stress Scale (DASS-21)	Screening tool for depression, anxiety, and stress
4	Edinburgh Postnatal Depression Scale (EPDS)	Screening tool for postnatal depression
5	Posttraumatic Stress Disorder Checklist for DSM-5 (PCL-5)	Symptom checklist for posttraumatic stress disorder
6	Center for Epidemiologic Studies Depression Scale panel (CES-D)	Screening tool for depression
7	Behavior and Symptom Identification Scale (BASIS-24)	Screening tool for psychosis

The INSTRUMENT_ITEM table was populated with 98 descriptions of values sourced from the instruments. Whenever feasible, these values were mapped to corresponding codes from the existing OMOP vocabulary set, enhancing the dataset's comprehensiveness and interoperability.

**Figure 9:**
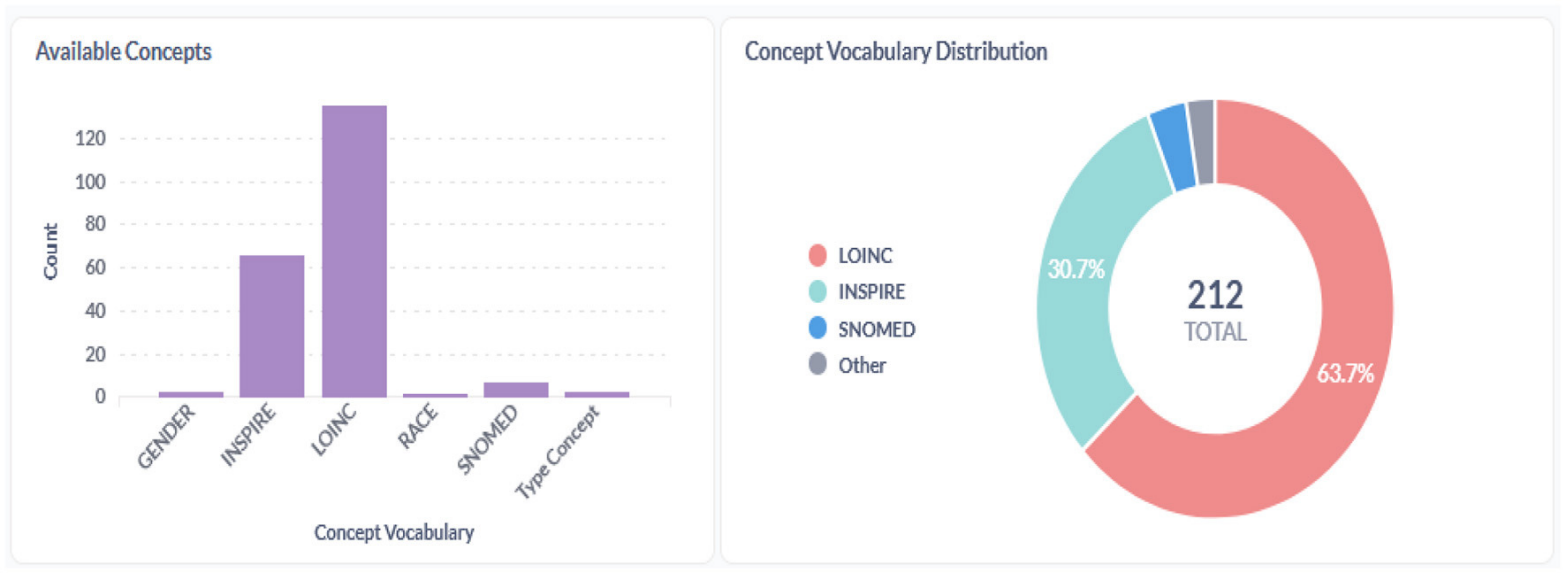
Concepts within the INSPIRE staging database.

**Figure 10:**
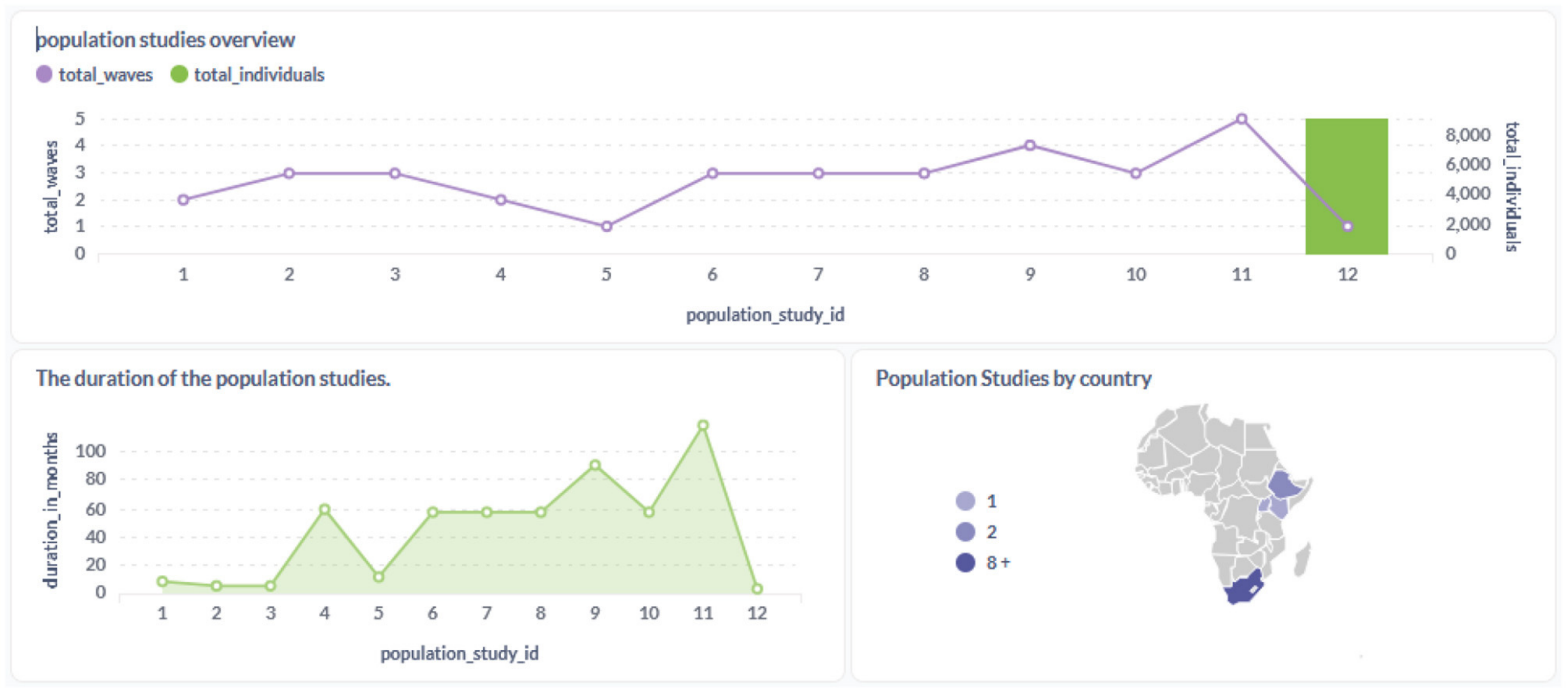
Overview of the population studies and metadata in the SD.

**Figure 11:**
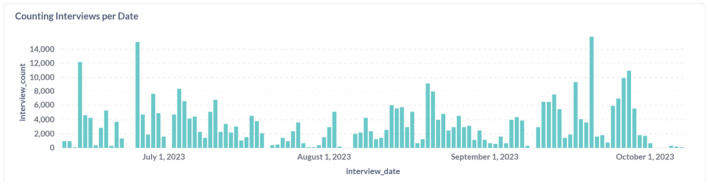
Count and distribution of interviews conducted in the first wave in Iganga.

Figures 9–11 provide insight into the concepts populated within the staging database. In addition to the standard OMOP vocabularies, the figures accommodate the local INSPIRE vocabulary utilized as an alternative for any missing corresponding codes in the OMOP vocabulary set.

The staging database was developed to meet the following model conditions.

#### 5.3.1 Manage complexity

A snowflake schema was designed to manage the complexity of hosting observations and measurements related to individuals, their demographics, resident episodes, household changes over time, interviews within waves, and instruments capturing facts. Due to its temporal complexity, the snowflake schema evolved into a more intricate structure with sub-dimensions. This database structure allows for the detailed representation of patient trajectories over time, with each table dedicated to capturing distinct aspects of the data, such as patient demographics, clinical assessments, and follow-up visits. Using a snowflake schema simplifies schema design in parts of the database and facilitates analytical queries, allowing efficient data analysis across multiple dimensions with the use of foreign keys.

#### 5.3.2 Establish relationships

Relationships between tables are explicitly defined using primary and foreign keys, ensuring that data across the database is interconnected. For example, a specific patient ID, serving as the primary key, can be linked to an assessments table as a foreign key, allowing for a direct connection between each assessment and the corresponding patient. These relationships enable the execution of complex queries spanning multiple tables, facilitating the extraction of valuable insights related to the progression of mental health conditions, treatment outcomes, and more.

#### 5.3.3 Facilitate normalization

The database employs normalization principles up to the third standard form (3NF) to reduce data redundancy and ensure data integrity. This involves organizing data into tables so that each one represents a single concept or entity type, with each column containing indivisible values and each row representing a unique instance of the entity type. Normalization eliminates duplicate data, making the database more efficient and easier to maintain, and helps to enforce data consistency using constraints and validation rules.

#### 5.3.4 Ensure scalability

The database was designed to easily add new tables and fields as new mental health assessment tools and data collection methodologies emerge. It utilizes modular design principles for seamless integration of new modules or components. The database management system features performance optimization tools such as indexing, partitioning, and data compression to handle increasing data volumes. Key fields, including primary and foreign keys, are indexed for efficient data retrieval, crucial for handling large longitudinal study datasets.

The Figure 10 presents an overview of population studies uploaded to the staging database.

## 6 Discussion

The central problem addressed here is the lack of standardized, interoperable, and comprehensive mental health data within the dynamically diverse settings of Africa., which significantly impedes the development of refined interventions. Creating the staging database, designed to encapsulate and streamline the meta(data) of longitudinal mental health studies, marks a pivotal advancement in data harmonization and standardization. Following the snowflake schema model, the database structure is specifically tailored to accommodate the complexity of longitudinal datasets that capture the multifaceted nature of mental health conditions over time.

The study acknowledges that perceiving health and illness as outcomes of multifaceted systemic interactions falls short of offering a comprehensive strategy for characterizing and statistically assessing such complexities. The standardized representation of mental health data facilitates comprehensive analysis, enhancing the ability to draw meaningful insights. This contributes significantly to the academic discourse on data harmonization and interoperability, demonstrating the practical application of advanced data science methodologies in global health research. Additionally, creating a detailed vocabulary set that incorporates OMOP and INSPIRE-specific terms exemplifies the project's adaptability and capacity to handle diverse data types and values, further underscoring its contribution to the field. More specifically, the staging database was created to tell the story of longitudinal studies that consist of field visits to households where individuals come and go over time. In the INSPIRE Mental Health Project with both secondary and primary data, the process specifies mainly the repeated use of standardized scales, including questionnaires, and, in the case of death, the Verbal Autopsy (World Health Organization).

The staging database orchestrates the visits and the panels administered from one visit to the next. To tell this story, the staging database took the form of a snowflake schema with dimensions for a study, an individual, their resident episodes, an interview, its instrument items, and a concept dimension used to tag questions and answers semantically and/or variables and values for downstream use in standard data models like OHDSI's OMOP CDM and in the analysis workbenches that run with these common data models like OHDSI's ATLAS, with some FACT tables aligning with both the INDEPTH data model for demographic surveillance systems and the DDI Lifecycle (Data Documentation Initiative-CDI).

However, the process of adapting longitudinal data to the staging database was met with several challenges. Among them was the need to maintain the longitudinal integrity of the data while fitting it into a model traditionally optimized for episodic healthcare data. Through iterative design and testing, a methodology was refined to preserve the dataset's temporal components within the model. This involved the development of the proposed framework for data transformation and representation, ensuring that the time-sequenced nature of the information remained analytically valuable. The results from the process resonate with the study conducted by Data Documentation Initiative and Ahmadi et al. (2022), who sought to tackle the complexities of standardizing electronic health records (EHRs) for enhanced clinical outcome predictions and patient care improvements. EHR-QC's creation addresses the heterogeneity and irregularities commonly found in EHR data, facilitating its conversion to a standardized format. The parallel between the two methodologies for longitudinal data adaptation and EHR-QC underscores a shared commitment to advancing digital health research. Both endeavors aimed to catalyze medical research by providing robust frameworks for the practical, data-driven generation of widely applicable data insights.

Therefore, the results from the staging database development underscore its success in achieving a synergistic convergence of complex longitudinal mental health data with a globally recognized data model. This has paved the way for insightful research and evidence-based policymaking in mental health.

### 6.1 Strengths, limitations, and implications for practice

Examining the staging database's strengths involves identifying its effective organization and data integration capabilities. Recognizing its limitations requires acknowledging potential scalability issues or data compatibility challenges. Assessing the practical implications involves understanding how the staging database can enhance data management, streamline analysis, and facilitate collaboration between population and clinical data. Exploring longitudinal datasets highlights opportunities for tracking trends, understanding treatment outcomes, and informing decision-making processes.

#### 6.1.1 The staging database

The staging dataset in this study plays a crucial role in integrating and preparing data for downstream tasks by serving as a buffer for data operations without compromising the integrity of the sources. Its focus on transforming, cleaning, validating, and standardizing datasets ensures quality, reliability, scalability, and usability for analytics, reporting, and visualization. Acting as a central repository, the staging database effectively stores various mental health assessment tools, codes, mappings, and vocabularies. The adoption of a warehouse snowflake schema promotes standardization, scalability, and sustainability, thereby facilitating interoperability and harmonization across datasets. Moreover, this setup enables seamless data migration to the OMOP CDM, integrating with OHDSI tools for advanced analytics and visualization. This methodological framework advances mental health data integration and offers a scalable and generalizable model for other researchers, fostering improved data quality and supporting extensive research collaborations (Benzler et al., 1998).

#### 6.1.2 Use of longitudinal datasets

Using longitudinal datasets in mental health research is invaluable, but it does pose certain limitations. Unlike more straightforward cross-sectional data, longitudinal datasets require complex relational structures representing temporal dependencies. Due to ongoing data collection, the staging database may have incomplete representation, resulting in missing waves or data. Furthermore, missing data is expected in longitudinal datasets due to prolonged collection periods or loss of follow-up, necessitating the approximation of interview dates for loading into the staging database. Additionally, manually inputting metadata across different data collection periods is time-consuming. To effectively address these challenges and ensure scalability and adaptability in global mental health research, refining ETL methodologies is essential (GitHub link: https://github.com/APHRC-DSE/Staging-Database-Integrating-Longitudinal-Mental-Health-Data.git).

## 7 Conclusion

Key achievements of this undertaking include the successful mapping of primary and secondary datasets into the staging database. This integration enhances the OMOP CDM's capacity for handling longitudinal mental health research parameters, thereby widening its applicability beyond its conventional use for cross-sectional data analysis. The enriched framework allows for a more nuanced interpretation of trends and patterns within the African demographic, demonstrating marked improvements in the staging and subsequent analysis of mental health data. This progression research underlines a shared vision with the findings of Ahmadi et al. (2022), whose cancer prediction investigations have underscored the critical role of standardized, harmonized data in the evolution of personalized healthcare and early diagnostic methodologies. The study highlighted that with the 2020 addition of a genomic vocabulary extension, OMOP CDM now operates at the vanguard of standardizing data analyses, which holds considerable promise for AI-centric predictive models. OHDSI first introduced episodes and survey instruments into OMOP CDM 5.4, which has proven to be experimental in giving the INSPIRE team history about how to map its longitudinal studies to the staging database.

Furthermore, the database's architecture was enriched by including place-based exposures and social determinants of health, extending the resident episodes we can capture using a combination of the DDI Lifecycle and the INDEPTH specification (The Hyve; Benzler et al., 1998). By doing so, the database provides a snapshot of the mental health condition and situates it within the broader community and environmental factors. This comprehensive approach allows for considering a wide array of variables downstream in OMOP and the OHDSI data analysis workbench that influence mental health outcomes, making the database a potent tool for both retrospective and prospective studies. Additionally, the staging database serves as a central repository for various mental health assessment tools, codes, mappings, and vocabularies, facilitating comprehensive analysis by integrating population and clinical data to provide a holistic view of mental health trends and intervention strategies. This systematic approach sets a new standard for mental health research, offering a scalable and generalizable model for researchers and institutions.

## 8 Future

**Figure 12:**
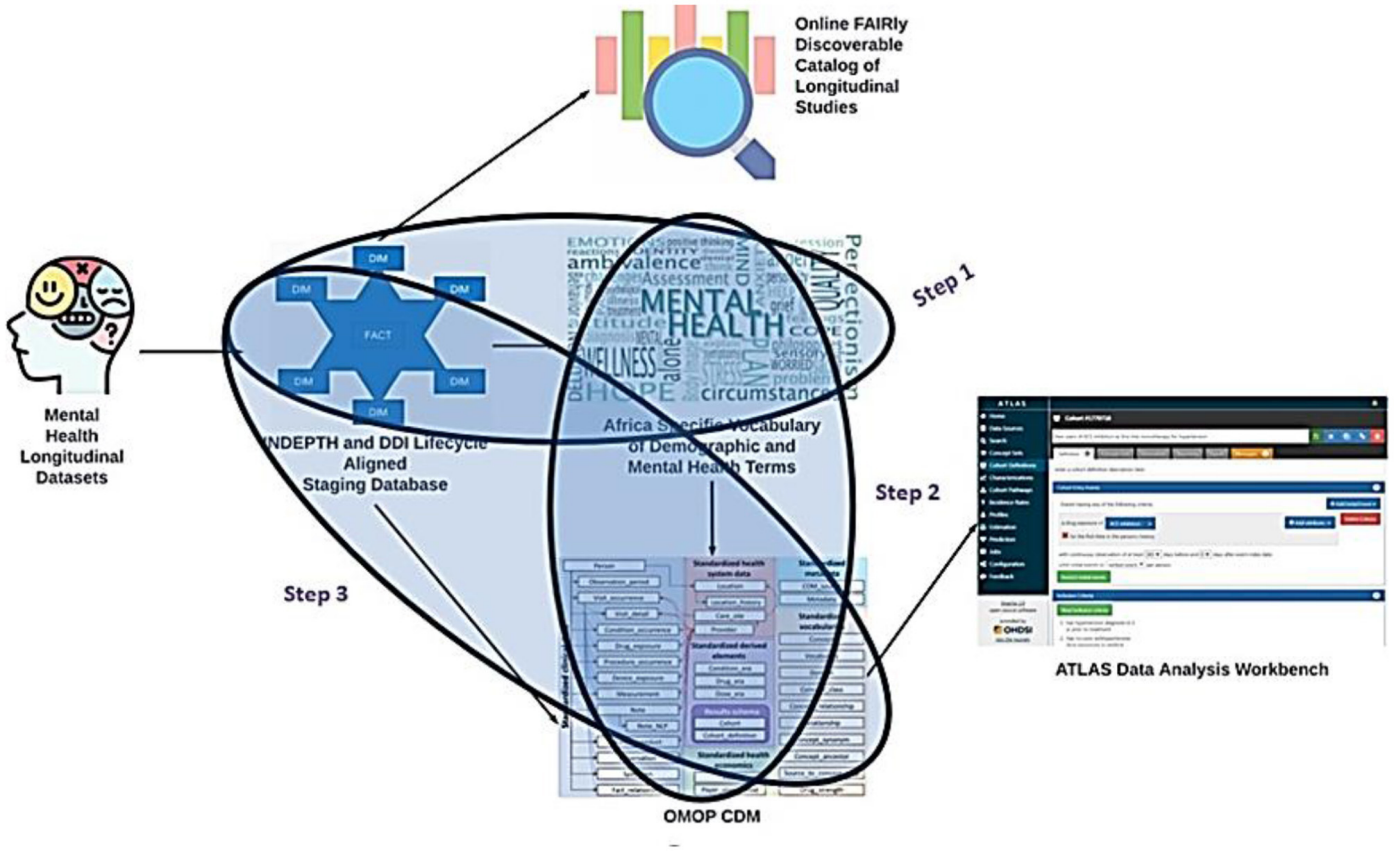
The mapping process.

This section elaborates on the following plan in three distinct steps. This will aim to enhance the integration and standardization of mental health data within the OMOP CDM framework, as illustrated in Figure 12.

### 8.1 Mapping longitudinal studies to OMOP CDM

#### 8.1.1 Building a custom vocabulary

The first step involves utilizing the staging database as input to construct a custom vocabulary by the MH INSPIRE team. This tailored vocabulary encompasses various instruments, including the psychosis screening tools and other pertinent metrics not currently covered not currently covered by standard vocabularies such as LOINC (Logical Observation Identifiers Names and Codes), which is a universal code system for identifying health measurements, observations, and documents. This vocabulary creation aims to bridge the gap between existing standardized terminologies and the specific nuances of mental health assessments.

#### 8.1.2 Integration into OMOP CDM vocabulary tables

In the second step, the MH INSPIRE team will incorporate the newly developed custom vocabulary into a dedicated instance of the OMOP CDM vocabulary tables, tailored explicitly for the INSPIRE MH Project. This integration ensures that the custom vocabulary becomes integral to the standardized terminology repository within the OMOP CDM framework, enabling seamless data interoperability and analysis across different data sources and projects.

#### 8.1.3 Referencing the custom vocabulary in ETL development

Finally, in the third step, the MH INSPIRE team will reference the custom vocabulary within the ongoing development of the Extract, Transform, Load (ETL) process. This ETL process is designed to generate OMOP observations and measurements within the bespoke mental health instance of the OMOP CDM. By incorporating the custom vocabulary into the ETL workflow, the team ensures that the generated observations and measurements accurately capture and represent the intricacies of mental health data defined by the custom vocabulary.

## Data Availability

The datasets presented in this study can be found in online repositories. The names of the repository/repositories and accession number(s) can be found in the article/supplementary material.
